# Cost effectiveness review of text messaging, smartphone application, and website interventions targeting T2DM or hypertension

**DOI:** 10.1038/s41746-023-00876-x

**Published:** 2023-08-18

**Authors:** Ruben Willems, Lieven Annemans, George Siopis, George Moschonis, Rajesh Vedanthan, Jenny Jung, Dominika Kwasnicka, Brian Oldenburg, Claudia d’Antonio, Sandro Girolami, Eirini Agapidaki, Yannis Manios, Nick Verhaeghe, DigiCare 4You, Natalya Usheva, Violeta Iotova, Andreas Triantafyllidis, Konstantinos Votis, Florian Toti, Konstantinos Makrilakis, Chiara Seghieri, Luis Moreno, Sabine Dupont, Leo Lewis, Djordje Djokic, Helen Skouteris

**Affiliations:** 1 Interuniversity Center of Health Economic Research (ICHER), department of Public Health and Primary Care, Ghent University, Ghent, Belgium; 2 Department of Food, Nutrition and Dietetics, School of Allied Health, Human Services and Sport, La Trobe University, Melbourne, Australia; 3 Institute for Physical Activity and Nutrition, Deakin University, Geelong, Victoria Australia; 4 Department of Population Health, NYU Grossman School of Medicine, New York, USA; 5 Maternal, Child and Adolescent Health Program, Burnet Institute, Melbourne, Australia; 6 NHMRC CRE in Digital Technology to Transform Chronic Disease Outcomes, Baker Heart and Diabetes Institute, Melbourne, Australia; 7 Faculty of Psychology, SWPS University of Social Sciences and Humanities, Wroclaw, Poland; 8 Meteda S.r.l., Roma, Italy; 9 Ministry of Health, Athens, Greece; 10 Department of Nutrition and Dietetics, School of Health Science and Education, Harokopio University, Athens, Greece; 11 Institute of Agri-food and Life Sciences, Hellenic Mediterranean University Research Centre, Heraklion, Greece; 12 Medical University of Varna, Varna, Bulgaria; 13 Centre for Research and Technology Hellas – Information Technologies Institute, Hellas, Greece; 14 University of Medicine, Tirana, Albania; 15 National and Kapodistrian University of Athens, Athens, Greece; 16 Sant’Anna School of Advanced Studies, Pisa, Italy; 17 Universidad de Zaragoza, Zaragoza, Spain; 18 International Diabetes Federation European Region, Brussels, Belgium; 19 International Foundation of Integrated Care, Schiphol, The Netherlands; 20 Privanova, Paris, France; 21 Monash University, Melbourne, Australia

**Keywords:** Health care economics, Hypertension, Metabolic disorders, Type 2 diabetes, Pre-diabetes

## Abstract

Digital health interventions have been shown to be clinically-effective for type 2 diabetes mellitus (T2DM) and hypertension prevention and treatment. This study synthesizes and compares the cost-effectiveness of text-messaging, smartphone application, and websites by searching CINAHL, Cochrane Central, Embase, Medline and PsycInfo for full economic or cost-minimisation studies of digital health interventions in adults with or at risk of T2DM and/or hypertension. Costs and health effects are synthesised narratively. Study quality appraisal using the Consensus on Health Economic Criteria (CHEC) list results in recommendations for future health economic evaluations of digital health interventions. Of 3056 records identified, 14 studies are included (7 studies applied text-messaging, 4 employed smartphone applications, and 5 used websites). Ten studies are cost-utility analyses: incremental cost-utility ratios (ICUR) vary from dominant to €75,233/quality-adjusted life year (QALY), with a median of €3840/QALY (interquartile range €16,179). One study finds no QALY difference. None of the three digital health intervention modes is associated with substantially better cost-effectiveness. Interventions are consistently cost-effective in populations with (pre)T2DM but not in populations with hypertension. Mean quality score is 63.0% (standard deviation 13.7%). Substandard application of time horizon, sensitivity analysis, and subgroup analysis next to transparency concerns (regarding competing alternatives, perspective, and costing) downgrades quality of evidence. In conclusion, smartphone application, text-messaging, and website-based interventions are cost-effective without substantial differences between the different delivery modes. Future health economic studies should increase transparency, conduct sufficient sensitivity analyses, and appraise the ICUR more critically in light of a reasoned willingness-to-pay threshold.

**Registration:** PROSPERO (CRD42021247845).

## Introduction

An estimated 537 million people, of which 44.7% undiagnosed, are nowadays living with diabetes mellitus worldwide. Type 2 Diabetes Mellitus (T2DM), formerly known as non-insulin-dependent or adult-onset diabetes, accounts for 90% of the population with diabetes^1^. Additional estimates indicate 541 million people having impaired glucose tolerance and 319 million having impaired fasting glucose levels, otherwise defined as prediabetes^1^, and are as such at increased risk to progress to T2DM^2^.

T2DM often occur together with hypertension^3^, another major risk factor for cardiovascular disease. Regarding its prevalence, hypertension affects around 1.28 billion or one-third of all adults between 30 and 79 globally, with 46% of them being unaware of the condition (https://www.who.int/news-room/fact-sheets/detail/hypertension). Prevalence is slightly decreasing in high-income countries but it is still on the rise in low- and middle-income countries^4^. An additional quarter to half of the global adult population is presumed to have pre-hypertension, defined as high normal office systolic and diastolic BP^5^.

Both diabetes and hypertension impose a substantial burden on healthcare budgets with 11.5 and 10% of global health expenditures spent on diabetes^1^ and high BP^4^, respectively. It is encouraging that preventive measures targeting modifiable lifestyle risk factors could result in substantial health and economic gains^1,6^. Lifestyle interventions focusing on diet modifications and increased physical activity have been proven effective in reducing HbA1c-levels and BP values^7^, and despite some discrepant results and varying study quality, these lifestyle interventions were found to be cost-effective as well^8–10^. Lifestyle interventions are thus valid strategies but the cost-effectiveness of various programmes and their drivers still need to be better documented. Staff labour cost is such an important driver, accounting for the larger part of lifestyle intervention costs and could thus be a potential target for improving cost-effectiveness^11^, for instance with the use of digital health interventions.

There is increasing evidence of digital health interventions as a practical, low labour and low cost delivery mode^12^ that can foster clinical-effectiveness and cost-effectiveness of such lifestyle measures. The potential is great since 92% of the global population uses a mobile phone^13^. The clinical effectiveness of digital health in diabetes and hypertension management has been confirmed^14–20^ but limited cost-effectiveness data have been referred to as one of the major barriers for widespread implementation^21^. Yet, Iribarren et al.^22^ reported that 75% of full economic studies (i.e., a comparison of both costs and health consequences of two or more alternatives) in a broad range of conditions found cost-effective or cost-saving results for mobile health solutions. Specifically, for T2DM, previous systematic reviews focused mainly on partial economic evaluations (i.e., single programme description of both costs and health consequences, or cost description/analysis of one or more alternatives)^23,24^. Nevertheless, the few full economic evaluations showed favourable results for digital health intervention modes such as phone/video calls, Short Message Service (SMS), and telemonitoring^23^.

The current study aims to systematically review full health economic evaluations of digital health interventions targeting the prevention and treatment of T2DM and/or hypertension in adults with (pre)diabetes and/or (pre)hypertension: smartphone applications, text-messaging, and websites are the subject of investigation^19,20^. This systematic review extends previously published systematic reviews that were mainly based on partial economic evaluations. Although such evaluations can provide some trends in this field, it is critically important in rapidly evolving research fields, such as digital health, to synthesize and report on more comprehensive economic evaluations^25^. The current systematic review focuses on three specific digital technology modes and discusses the health economic evidence for each mode separately. Finally, this review puts emphasis on the methodological quality appraisal, with a focus on making informed recommendations to improve methodological quality.

## Results

### Study selection

**Fig. 1:**
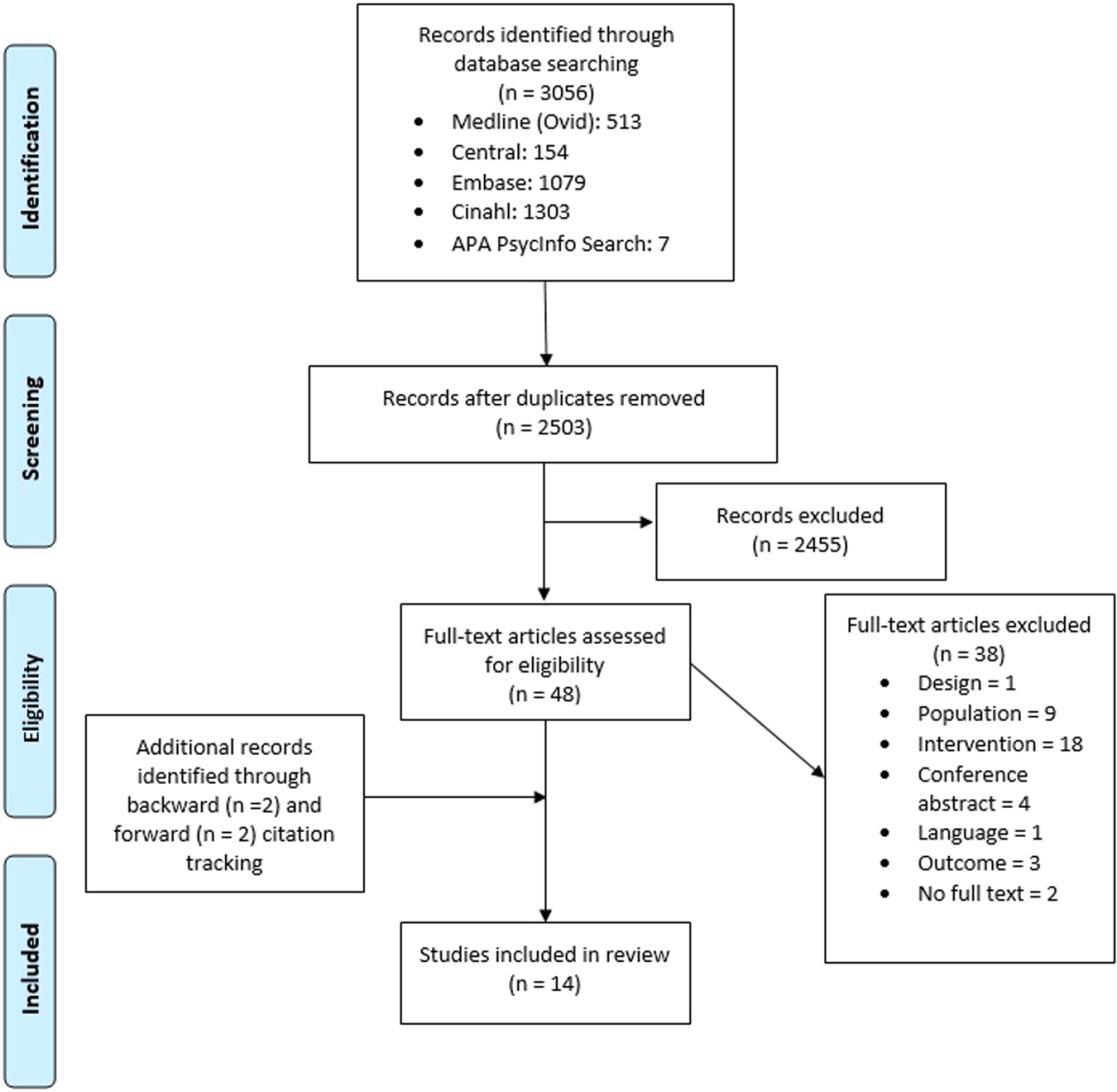
PRISMA flow diagram.

The process of study identification, screening, and inclusion is displayed in the PRISMA flow diagram in Fig. 1. From 3056 studies (2503 after duplicate removal) identified through database searches, 14 studies evaluated the value for money of either website^26–30^, text-messaging^27,31–36^, or smartphone application interventions^29,37–39^. An overview of study characteristics, intervention details, and health economic outcomes can be found in Table 1. Supplementary note 1 shows excluded studies at full text screening with reasons. Augustovski et al.^31^ and Zhang et al.^36^ reported on the same trial but the former was a trial-based analysis while the latter was model-based to extrapolate costs and effects on the long-term.

**Table 1:** Evidence table.

	Study characteristics					Intervention			Health economic outcomes			
Study	Country	Patient population, sample size	Design & Time horizon	Perspective	Discount rate	Digital health intervention	In combination with other (digital) interventions?	Comparator	Incremental costs [converted to €, 2022]	Incremental effects	ICUR/ICER/ROI [converted to €, 2022]	Authors' conclusion
Augustovski et al. (2018) and Zhang et al. (2021)	Argentina	Hypertension IG: 743 CG: 689	Trial-based 18 months	Public healthcare system	5%	Text-messaging	- Case manager - Physician education	CAU	$, 2017: 140 [€126]	QALY: 0.042 SBP: -5.30 mmHG Hypertension control: 19%	$/QALY: 3299 [€2958] $/mmHG SBP reduction: 26.43 [€23.70] $/extra person under threshold: 721 [€646]	Cost-effective
			Markov-model Lifetime						$, 2017: 623 [€559]	QALY: 0.13	$/QALY: 4907 [€4400]	Cost-effective
Chen et al. (2016)	USA	Prediabetes IG: 997 CG: 1:1 match with historical cohort	Markov model 10 years	Healthcare payer	3%	Website-based intervention including a 16 week online training programme and online social network support communities	- Telemonitoring - Health coach - 24/7 telephone and online professional availability	CAU	$, 2015 at 3 y: -800 [-€739] $, 2015 at 5 y: -3330 [-€3076] $, 2015 at 10 y: -13,240 [-€12,230]	/	ROI at 3 y: 1.61 ROI at 5 y: 3.56 ROI at 10 y: 11.18	Cost-saving
Cunningham et al. (2022)	United Kingdom	T2DM IG: 2576 CG: 5:1 match	Markov model 10 years	Public healthcare system^1^	3.5%	Website and smartphone app for education and access to electronic personal health record for communication with professionals, patient involvement, and shared-decision making.	/	CAU	£, 2018: -84.71 [-€106.57]	QALY: 0.054	£/QALY: -1569 [-€1974]	Dominant
Derakhshandeh-Rishehri et al. (2022)	Iran	Non-insulin dependent T2DM IG: 35 CG: 35 CG2: 35	Trial-based	Patient perspective reported but conflicts with cost overview	/	Web/online nutrition education once a month with internet and WhatsApp training upfront	/	CG1: monthly group sessions with similar education content as IG CG2: CAU (although CAU was non-existing due to Covid-19)	$, 2021: IG vs CG1: -21,853 [-€18,067] IG vs CG2: 4971 [-€4110]	HbA1c: IG vs CG1: -0.17% IG vs CG2: -0.23%	$/% HbA1c reduction: IG vs CG1: dominant IG vs CG2: 21,613 [-€17,869]	Cost-effective compared to care as usual
Faleh Al-Mutairi et al. (2021)	Saudi Arabia	T2DM IG: 100 CG: 100	Retro-spective matched cohort study 4 months	Public healthcare system^1^	/	Text-messaging for education and medication management	- Telemonitoring - Teleconsultation	CAU (In-person care, which requires physical attendance at the integrated care clinic)	SAR,2020: 669 [€897]	HbA1c: -0.28%	SAR/% HbA1c reduction: 2373 [€1356]	Cost-effective^3^
Gilmer et al. (2019)	Mexico	T2DM IG: 102 CG1: 99 CG2: 99	Markov model Lifetime	Public healthcare system	3%	Text-messaging (and videos)	- Telemonitoring - Teleconsultation - Self-management training - Education of physicians and nurses	CAU+ - CG1: self-management training and education of physicians and nurses - CG2: monthly face-to-face follow-up and option to join a monthly peer support group	$, 2017: IG vs CG1: 212 [€190] IG vs CG2: 599 [€537]	QALY IG vs CG1: 0.05 IG vs CG2: 0.22	$/QALY IG vs CG1: 4299 [€3855] IG vs CG2: 2220 [€1991]	Cost-effective
Islam et al. (2020)	Bangla-desh	T2DM IG: 118 CG: 118	Trial-based 6 months	Limited public healthcare system	/	Text-messaging	/	CAU	BDT, 2013: 558 [€21]	QALY: 0.010 HbA1c: -0.64%	BDT/QALY: 55,800 [€2077] BDT/% HbA1c reduction: 871 [€32]	Cost-effective
Li et al. (2018)	United Kingdom	T2DM IG: 185 CG: 189	Trial-based 12 months	Public healthcare system	/	- Web: education, social network support, and ask the expert facility. - Text-messaging	/	CAU+ Access to comparator website containing basic information plus education on how to use the website.	£, 2014: 111 [€149]	QALY: 0.020 PAID: -1.9	£/QALY: 5550 [€7454] £/unit improvement on PAID scale: 58 [€78]	Cost-effective
Li et al. (2021)	China	T2DM IG: 130 CG: 85	Trial-based 12 months	Public healthcare system^1^	/	Smartphone app to provide peer support, patient education, telemonitoring and communication with care provider	/	CAU+ Three monthly examination of HbA1c, FBG, and P2BG levels to support medication adjustments. Patient education at each encounter.	CNY, 2019:^2^ -606 [-€149]	Prevalence difference in HbA1c control rate ( < 7%): 27.50%	CNY/patient with controlled HbA1c level: -2202 [-€540]	Dominant
McManus et al. (2021)	United Kingdom	Hypertension IG: 305 CG: 317	Trial-based 12 months	Public healthcare system^1^	/	Web education	- Telemonitoring - Education of healthcare workers - Up to 6 brief contacts to support behavioural changes	CAU+ Online patient leaflet for hypertension.	£, 2019:^2^ 38 [€47]	QALY: 0.002 SBP: -3.5 mmHg	£/QALY: not calculated as QALY difference was insignificant £/unit SBP reduction: 11 [€14]	Cost-effective^3^
Tsuji et al. (2020)	Japan	T2DM cohort	Markov model 20 years	Limited public healthcare system	4%	Smartphone app for telemonitoring and communication with care provider and family.	/	CAU	$, 2018:^2^ 3634 [€3187]	QALY: 0.11	$/QALY: 33,039 [€28,971] Simulated result which should be confirmed by future trial data	Cost-effective
Wong et al. (2016)	China	Prediabetes IG:54 CG:50	Markov model Lifetime	Public healthcare system	3%	Text-messaging	/	CAU+ Information booklets on (pre)diabetes and health behaviour.	$, 2011: -1020 [-€1006]	QALY: 0.071 Life years: 0.063	$/QALY: -14,371 [-€14,177] $/Life year: -16,196 [-€15,977]	Dominant
Zhang et al. (2020)	China	Hypertension IG: 101 CG1: 95 CG2: 87	Decision tree model 6 months	Patient	/	Smartphone app to provide patient education, guidance, telemonitoring, health agenda.	/	- CG1: CAU - CG2: CAU+ self-management	CNY, 2017: IG vs CG1: 105 [€27] IG vs CG2: 590 [€150] CG2 vs CG1: -485 [-€124]	QALY: IG vs CG1: 0.007 IG vs CG2: 0.002 CG2 vs CG1: 0.005	CNY/QALY IG vs CG1: 15,000 [€3825] IG vs CG2: 295,000 [€75,233] CG2 vs CG1: -97,000 [-€24,738]	Cost-effective compared to CAU but not compared to CAU+

^1^perspective not reported but authors’ judgement based on available information; ^2^reference year not reported so publication year minus two; ^3^no willingness-to-pay threshold reported or no valid argumentation given why the given willingness-to-pay threshold is justified; *CAU* care as usual, *CG* control group, *ICER* incremental cost-effectiveness ratio, *ICUR* incremental cost-utility ratio, *IG* intervention group. *QALY* quality-adjusted life year, *ROI* return-on-investment.

### Study characteristics

Included studies reflected a broad geographic distribution with one study conducted in North America^26^, one in Central America^32^, two in South America^31,36^, four in East Asia^34,37–39^, one in South Asia^33^, two in the Middle East^30,35^, and three in Europe^27–29^. Eight studies included people with T2DM^27,29,30,32,33,35,37,39^, two included people with prediabetes^26,34^, and four studies focused on people with hypertension^28,31,36,38^.

Five studies were within-trial analyses with a time horizon between 6 and 18 months and a public healthcare system perspective^27,28,31,33,39^, while one was a retrospective matched cohort study applying similar analytics^35^. The within-trial analysis of Derakshandeh-Rishehri et al.^30^ applied a patient perspective but this is disputable. One study used a decision tree-based model with a time horizon of 6 months and a patient perspective^38^. Five studies used a Markov model to estimate long-term (i.e., 10 years to lifetime) costs and effects based on clinical trial inputs, and whereby three applied a public healthcare system perspective^29,32,34,36^ and one a healthcare payer perspective^26^. Finally, there was one Markov-model study which did not directly stem from one particular implementation study (i.e., all input parameters were literature driven) and which applied a 20-year horizon^37^. All studies with a time horizon of more than 1 year applied discount rates for both future costs and health outcomes between 3 and 5%^26,31,32,34,36,37^.

### Interventions

Four studies evaluated the use of smartphone applications, one in people with hypertension^38^ and three in people with T2DM^29,37,39^. Smartphone applications were used for monitoring, treatment adaptation, and communication between patients and healthcare professionals (in Tsuji et al.^37^, also for communication with family). The smartphone applications in Li et al.^39^ and Cunningham et al.^29^ were also used for patient education. The smartphone application in Zhang et al.^38^ included a health agenda (i.e., reminders for follow-up). None of the four studies on smartphone applications included non-digital intervention features.

Seven text-messaging^27,31–36^ and five website-based studies^26–30^ were included. One intervention combined text-messaging and websites^27^, while five other interventions also comprised (non-)digital health modalities such as the implementation of a case manager or teleconsultation^26,28,31,32,35,36^. Text-messaging was used to encourage the adoption of healthier lifestyle behaviours by participants. The length of the intervention ranged from 16 weeks to 2 years, and the frequency of text messages could be as high as daily but it was not always reported. The website-based intervention component consisted of educational web pages and social network support groups, often in addition to teleconsultation, face-to-face follow-up, and/or telemonitoring.

Interventions were compared to care as usual^26,29–31,33,35–38^ or an enhanced version of care as usual (comprising self-management training, education, and/or physician training)^27,28,32,38,39^.

### Health outcomes

Ten studies reported on the cost per quality adjusted life year (QALY) as the primary health economic outcome^27–29,31–34,36–38^. Some studies reported clinical outcomes such as systolic blood pressure reduction^28,31^, HbAc1 reduction^30,33,35^, proportion of population reaching hypertension^31^ or glycemic control^39^, life years gained^34^, and points gained on the problem areas in diabetes control (PAID) scale^27^. The cost-minimisation study of Chen et al.^26^ reported on the return on investment.

### Quality appraisal

Table 2 shows the critical appraisal of selected studies for the evaluation of their quality. More than half of the included studies did not provide sufficient detail on the comparative alternatives (i.e., what does care as usual actually mean). Nine studies did not describe important costing aspects such as how the costs were measured or the sources of cost valuation^26,28–30,33,35,37–39^. A rather short time horizon was applied in more than half of the studies^27,28,30,31,33,35,38,39^ despite a long-time horizon being recommended in evaluating cost-effectiveness of chronic diseases to capture all relevant costs and effects. Moreover, all but one study^27^ did not provide sufficient argumentation for choosing another perspective to the societal one. Finally, only six studies reported both probabilistic sensitivity results plus another kind of sensitivity analysis such as threshold analysis or one-way sensitivity analysis on top of the point estimate results^27–29,31,32,36^.

**Table 2:** Quality appraisal with the CHEC-list.

	Augustovski (2018)	Zhang (2021)	Chen (2016)	Cunningham (2022)	Derakhshandeh-Rishehri (2022)	Faleh Al-Mutairi (2021)	Gilmer (2019)	Islam (2020)	Li (2018)	Li (2021)	McManus (2021)	Tsuji (2020)	Wong (2016)	Zhang (2020)	
Study population	0	0	1	1	1	1	0	1	1	1	1	1	1	1	79%
Competing alternatives	0	0	0	0	0	1	1	0	1	1	1	0	0	1	43%
Research question	0	0	0	1	1	0	0	0	1	1	1	0	0	0	36%
Study design	1	1	0	1	0	1	1	1	1	1	1	1	1	1	86%
Time horizon	0	1	1	1	0	0	1	0	0	0	0	1	1	0	43%
Perspective	0	0	0	0	0	0	0	0	1	0	0	0	0	0	7%
Costs: identification	1	1	1	1	0	1	1	0	1	1	0	0	1	1	71%
Costs: measurement	1	1	0	0	0	0	1	0	1	0	0	1	1	1	50%
Costs: value	1	1	0	0	0	0	1	0	1	0	0	1	1	0	43%
Outcomes: identification	1	1	NA	1	1	1	1	1	1	1	1	1	1	1	100%
Outcomes: measurement	1	1	NA	0	1	1	1	1	1	1	1	1	1	1	92%
Outcomes: value	1	1	NA	0	0	0	1	1	1	1	1	1	0	1	77%
Incremental analysis	1	1	NA	1	1	1	1	1	1	1	1	1	1	1	100%
Discounted	1	1	1	1	NA	NA	1	NA	NA	NA	NA	1	1	NA	100%
Sensitivity analysis	1	1	0	1	0	0	1	0	1	0	1	0	0	0	43%
Conclusions	1	1	1	1	0	1	0	0	1	1	1	0	1	1	71%
Generalizability	0	1	1	1	1	1	1	1	1	0	1	1	0	0	71%
No conflict of interest	1	1	0	0	1	1	1	1	1	1	0	0	1	1	71%
Ethics	1	1	0	1	0	0	0	1	1	0	1	0	0	1	50%
Quality score (%)	68	79	40	63	42	56	74	50	94	61	67	58	63	67	

More extensive item assessment instructions can be found in Appendix 3. *NA* not applicable.

### Data synthesis

**Fig. 2:**
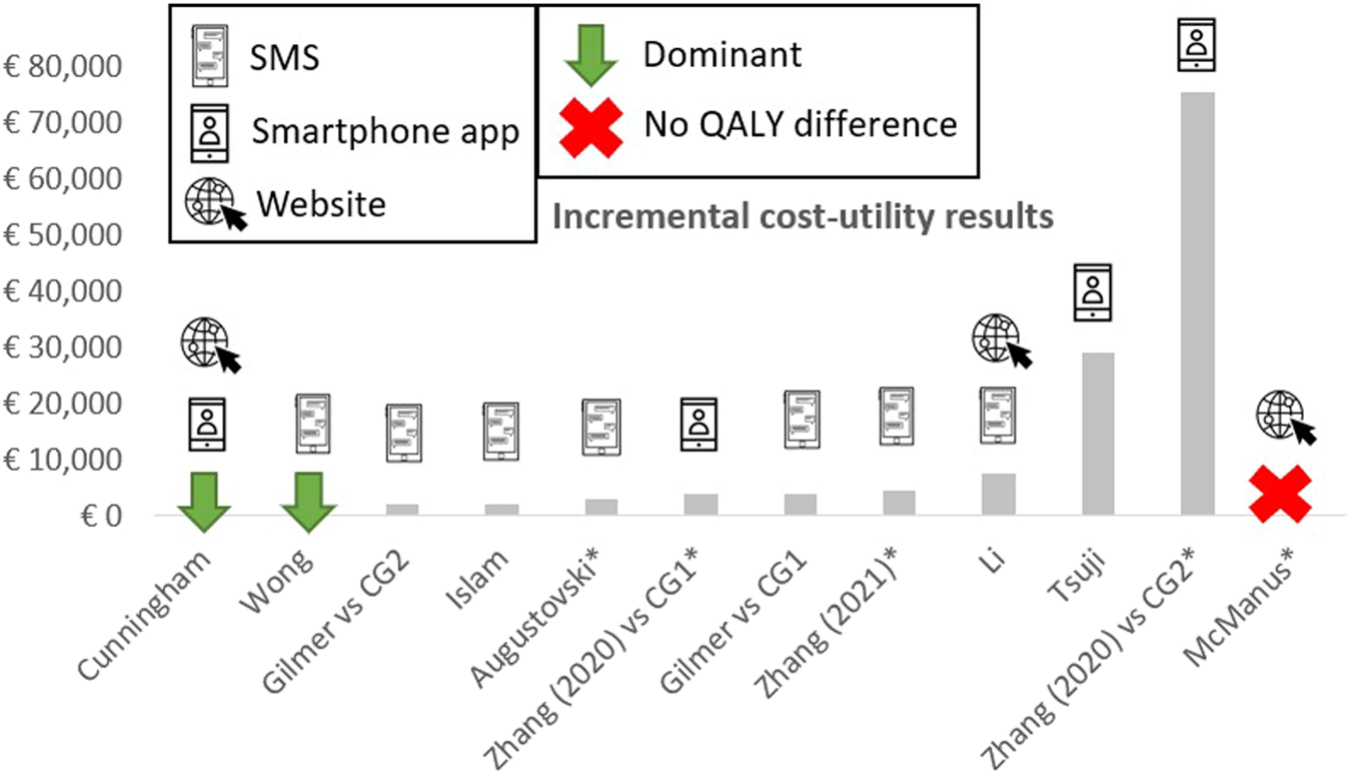
Incremental cost-utility results (ICUR) estimates of included studies. Note that McManus et al.^28^ did not calculate an ICUR as QALY difference was insignificant. ICUR estimates in Cunningham et al.^29^ and Wong et al.^34^ were dominant. CG control group. *: asterisk denotes studies targeting populations with hypertension; studies without an asterisk include people with (pre)diabetes.

Among the studies expressing results in QALYs, the ICURs varied between dominant (i.e., less costly and better health outcomes) and €75,233/QALY, with a median of €3840/QALY (interquartile range €16,179). One study did not find a QALY difference (Fig. 2). None of the three digital health intervention modes was associated with substantially better cost-effectiveness results than the others. Four out of fourteen studies (one on text messaging, two on mainly smartphone applications, and one on website-based education) reported cost-saving results^26,29,34,39^.

Smartphone applications were appraised by the studies’ authors as cost-effective^37,38^ or dominant^29,39^ compared to usual care. However, the cost-effective results in Tsuji et al.^37^ were associated with considerable uncertainty and should be confirmed by future trial data, as effectiveness data were simulated and the prediction model had been built on major assumptions. Li et al.^39^ did not report uncertainty analyses. Furthermore, the smartphone application in Zhang et al.^38^ was reported as not cost-effective compared to a self-management intervention: QALY gain was higher but at a considerable cost: a self-management strategy appeared to be the preferred strategy from a health economic perspective (Fig. 2).

Text-messaging alone, or in combination with other intervention aspects (such as teleconsultation, telemonitoring, case management), was found to be cost-effective^27,31–33,35,36^ or even cost-saving^34^. Although QALY gains were limited (ranging from a 0.01 increment per target person after 6 months in Islam et al.^33^ to a 0.22 increment per target person taking a lifetime horizon in Gilmer et al.^32^), the ICUR appeared to be robust in probabilistic sensitivity analysis^27,31,32,36^. This can be related to the low intervention costs since Islam et al.^33^ demonstrated that programme costs could at least be doubled while remaining cost-effective. Wong et al.^34^ even calculated that programme costs could be 50 times greater before the break-even point would be reached. Moreover, Li et al.^27^ argued that the health economic results of text-messaging can be even further improved by upscaling so that the cost per person decreases. Importantly, the ICUR in Gilmer et al.^32^ turned cost-effective only after 10–20 years, which was inconsistent with other studies that demonstrated cost-effectiveness in the short term^27,31,33^.

Website-based interventions appeared to be cost-effective^27,28,30^, dominant^29^, or cost-saving^26^, even though only a natural effect (i.e., a reduction in systolic blood pressure; the incremental number of QALYs was not significant) was found in the study by McManus et al.^28^. Yet scenario analysis, in which the intervention effect partly faded away, and probabilistic sensitivity analyses showed the results to be robust at given thresholds^26–29^.

Sensitivity and subgroup analyses were limited in most studies, which restricts the identification of cost-effectiveness drivers. First, Augustovski et al.^31^ reported on patient baseline characteristics: the intervention appeared to have greater value for money in populations of younger age, subjects with higher cardiovascular risk, higher body mass index, and women. The gender difference has been reported by Cunningham et al.^29^ as well.

Secondly, intervention aspects influenced the ICER/ICUR as well. A less intensive so less costly intervention following lower treatment adherence was reported by Augustovski et al.^31^, thus being indicative of better cost-effectiveness although the observed differences were not statistically significant. Meanwhile drop-out rates did not impact the ICER/ICUR in Wong et al.^34^. Costs were important drivers of cost-effectiveness in other studies as well^33,38^.

Third, modelling assumptions was the third and most investigated pillar of what drives cost-effectiveness results. The value for money improved with longer time horizons^26,32^, and the impact of transition probabilities, utility values, and discount rate on the ICUR were mixed^34,37,38^.

Whether digital health interventions targeting (pre)T2DM versus hypertension populations resulted in different cost-effectiveness outcomes, is difficult to assess because only three studies targeted populations with hypertension. However, it seems that digital health interventions targeting (pre)T2DM populations showed consistently positive cost-effective results^26,27,29,30,32–35,37,39^, while cost-effectiveness results in hypertension populations were more mixed^28,31,36,38^.

Whereas six studies evaluated one particular digital health mode, there were two studies that combined two of the digital health modes under investigation^27,29^, two studies where the digital health mode was part of a broader digital intervention including telemonitoring^26,35^, and four studies (three interventions) where the digital health mode was part of a broader health system intervention including digital and non-digital components^28,31,32,36^. Website-interventions, text messaging, and smartphone applications were complemented by, or were seen as a complement of, other intervention components in four out of five, four out of six, and one out of four times, respectively. Gilmer et al.^32^ and Zhang et al.^36^ evaluated two of the broader health system interventions and found relatively higher health effects (0.22 and 0.13 QALYs, respectively) compared to stand-alone interventions. Note that these two studies applied a long-term perspective, contrary to McManus et al.^28^ who evaluated a broad health system intervention and who found only a systolic blood pressure reduction on the short-term but no QALY improvement.

## Discussion

This review aimed to synthesize the available health economic evidence of digital health interventions in populations with or at risk of T2DM and/or hypertension. Digital health interventions were restricted to smartphone applications, text-messaging, and website-based interventions. The three digital health intervention modes were found to be cost-effective or cost-saving compared to care as usual and, most of the time, to enhanced care as usual too. Median ICUR of cost-utility studies was low with €3840/QALY.

Recent meta-analyses from our team have shown the three digital health interventions to be equally effective in reducing BP in adults with hypertension, while text-messaging and smartphone application interventions were associated with increased improvements in glycaemic control compared to website-based interventions in adults with T2DM^19,20^. However, increased effects did not always offset additional costs: when comparing the three digital intervention modes with (enhanced) care as usual, our analysis did not show a strong preference in terms of cost-effectiveness for one particular mode.

Digital health interventions seem to be consistently cost-effective in populations with (pre)T2DM but not in populations with hypertension. One possible explanation could be that the cost-effectiveness of implementing a digital health mode depends on the perceived severity of a condition and hence the urge to act upon. Hypertension is so widespread that some might perceive it merely as a risk factor instead of a disease^40^, so patients and professionals could be less motivated to do something about it. For example, New Zealand does not have hypertension guidelines but bases its care recommendations on a cardiovascular risk score^41^. Moreover, a global consensus definition of hypertension is lacking (see for example the definitions of different leading organisations: https://tinyurl.com/whohyp, https://tinyurl.com/cdchyp, https://tinyurl.com/mayhyp, https://tinyurl.com/nhshypdef). Smartphone applications, websites and text-messaging may have a significant clinical impact on BP, but there are possibly other approaches or other health objectives that better justify the money invested. This remains to be tested as the health economic evidence of smartphone apps, text-messaging, and website-based interventions in populations with hypertension remains very limited.

Among other process evaluation constructs, adherence and reach are two important ones with a major impact on digital health interventions’ cost-effectiveness^42^. Patients who adhere with a smartphone application showed, for instance, better medication adherence^43^. However, high drop-out rates of 40% (95% CI 16–63%) in RCT’s testing smartphone applications have been demonstrated as well^44^. It has been suggested that attrition could, for instance, be reduced by using user feedback to enhance user experience, by enabling the possibility for users to contact health professionals (a so-called hybrid model), by focusing on self-management skills, by increasing health literacy, and by combining smartphone applications with internet or telehealth solutions^44^.

Differentiating between primarily digital health interventions and primarily health system interventions with a digital component is warranted. Our results suggest that health system interventions might have the potential to gain more health effects on the long-term compared to a stand-alone digital health mode intervention, although current evidence is limited and mixed. However, Augustovski et al.^31^ suggested better cost-effectiveness when the intervention was less intense. Although their statement should be interpreted cautiously because of overlapping confidence intervals between the different intervention intensities, these observations might be in line with the results of a meta-analysis on drop-out rates of exercise interventions that demonstrated a higher likelihood of drop-out in more intensive interventions^45^. Therefore, future interventions should carefully consider which features needs to be combined, knowing that more intervention features could improve effectiveness but a too intense intervention may also increase complexity of use thus having a detrimental impact on both drop-out and cost-effectiveness. Participant input via co-design may be of a help from the evidence gathering to the real-world testing stage^46^.

Given that there are hundreds of millions of people with or at risk of T2DM and hypertension, it is important to keep an eye on the scalability and budget impact of a new programme^42^. Whereas clinical effectiveness on the individual level can be optimised by adding possible intervention components to tailor care, less elaborated programmes may have a higher reach resulting in more population benefit within a closed budget. This could be of particular importance to digitally less-developed countries where digital interventions might be relatively more expensive. Scrutinizing the optimal intervention dose in different health systems including digitally less-developed countries is therefore paramount. Our results indicate for instance that text-messaging is appraised as cost-effective across studies, either in combination with other intervention features or not. Self-monitoring can also be a very powerful strategy to improve cost-effectiveness as well. It might therefore be an option to integrate such functionalities in smartphone applications.

Our quality appraisal demonstrated important methodological shortcomings. Based on these, our four key lessons for future health economic evaluations of digital health interventions are:

1. Health economic results can only be appraised correctly with an elaborate research question and sufficient context. The competing alternatives under investigation – care as usual in particular – should be detailed. The study’s perspective should be justified and the applied time horizon should capture relevant long-term costs and effects of preventive measures^9,11^. In this regard, it is important to stress the cost-effectiveness results of digital therapeutics despite the sometimes quite short time horizons applied.
2. Transparency is pivotal when reporting applied costs: which costs have been included exactly (which refers to the perspective) and how these were measured and valued should be stated.
3. Health economic evaluations of digital health interventions often come with data uncertainty and assumptions. One-way and probabilistic sensitivity analyses are at least needed to address these uncertainties, preferably in different subgroups. Following key lesson 1 on applying an appropriate long time horizon, it is pivotal to scrutinize the impact of the intervention effect’s sustainability on the health economic outcome, especially given the high attrition and dropout rates in for instance app-based interventions^44^.
4. An ICUR does not have an intrinsic value and should always be evaluated in light of a willingness-to-pay threshold. Most included studies applied a threshold value of one to three times the gross domestic product (GDP) per capita, as recommended by the World Health Organisation^47,48^, but critics argued that a more conservative threshold of ±50% the GDP per capita would better capture opportunity costs^48,49^. Note that, given such a conservative threshold, most cost-effectiveness estimates of digital health interventions remain cost-effective. Furthermore, some studies applied natural units (e.g., cost per percentage HbA1c reduction). For instance, Derakhshandeh-Rishehr et al.^30^, Faleh Al-Mutairi et al.^35^ and McManus et al.^28^ reported an increase in health effect at an increased cost and stated the result was cost-effective although no willingness-to-pay threshold or valid argumentation for the applied willingness-to-pay threshold was reported, respectively.

These key lessons should be considered in future research. Such studies should also strive to address evidence gaps in the field. Head-to-head studies are definitely needed to determine the digital health mode with the best value for money in different subgroups operating within a particular health system. The uncertainty associated with long-term health economic evaluations can be reduced by designing trials with longer clinical follow-up periods so the sustainability of the intervention effect can be modelled more precisely. Moreover, budget impact estimates are truly relevant for policy makers given the high prevalence of T2DM and hypertension, while uptake and attrition rates should also be taken into consideration as they can also have a significant effect on the costs.

The most important strengths of this review are the complementarity with previously published meta-analyses^19,20^ scrutinizing the effectiveness of the three digital health intervention modes, and the thorough quality appraisal resulting in several key lessons for health economic research.

However, this systematic review also has limitations. First, the adult filter is not consistent between the five searched databases. In Medline, the adult filter is >19 years of age, whereas for EMBASE and PsycINFO it is >18. However, the proportion of the population with T2DM or hypertension at that age is small^50,51^. Second, studies on people with or at risk of T2DM or hypertension were included but the small number of studies impeded appropriate subgroup analyses. What may work in one population may not work in another. Third, only English articles were included and this may limit our conclusions, especially since T2DM and hypertension prevalence are high in large non-English-speaking countries such as India^52^ and China^53^. However, included studies from the Americas, Europe, Asia, and the Middle-East reflected a geographically and demographically diverse population. Fourth, digital health solutions in five website-based or text-messaging studies have been augmented with other intervention features such as healthcare professional education, telemonitoring and/or (tele)consultations. It is therefore not clear whether the intervention effect arises due to these additional intervention features or due to the digital intervention component. Fifth, the number of full health economic papers remain scarce, especially compared to the accumulating amount of clinical effectiveness evidence, and the results of Tsuji et al.^37^ are based on disputable assumptions. Because of the low number of included papers, additional analyses of the impact of study quality on results were not conducted. Sixth, no head-to-head health economic studies of the three digital intervention modes were found. Seventh, health economic studies might be subject to multiple sources of publication bias including a publication bias in first health outcome publications and next economic publications. Funnel plotting to investigate possible publication bias was not an option in this study but Moschonis et al.^19^ and Siopis et al.^20^ demonstrated respectively a small and non-existing publication bias in our health outcome reviews. It is of course still possible that a publication bias favoring cost-effective or cost-saving results remains in economic publications, especially given the suboptimal reporting of sensitivity analyses^54^.

In conclusion, health economic evidence suggests that smartphone application, text-messaging, and website-based interventions are cost-effective and, in some cases, even cost-saving. It shows how challenging, but at the same time how possible, it can be to improve the health of the population while saving money.

While previous research demonstrated that the three digital health intervention modes were equally clinically-effective in adults with hypertension, and that text-messaging and smartphone application interventions worked significantly better than website-based interventions in adults with T2DM, no cost-effectiveness evidence was found supporting one particular digital health intervention mode over another. Moreover, text-messaging, smartphone application, and website-based interventions appeared to be consistently cost-effective in populations with (pre)T2DM, but not in populations with hypertension.

Based on the available evidence, policy makers and clinicians should make decisions on the most appropriate digital health interventions based on available budgets and well-defined health objectives. The high penetration rate of digital applications in diverse populations is a strength but it is pivotal to keep process evaluation constructs in mind. Key lessons for future health economic studies on how to design studies and report on the results are given. It is important to pay special focus on the context, report the costs included and how these were measured and valued, conduct sufficient sensitivity analyses, and appraise the cost-effectiveness result more critically in light of a reasoned willingness-to-pay threshold. Head-to-head studies are missing while this would enhance understanding and practice substantially. It is strongly recommended to consistently include a cost-effectiveness work package alongside clinical trials^55^.

## Methods

### Literature search

The protocol (PROSPERO CRD42021247845) and reporting of this systematic review were consistent with the 2020 PRISMA guidelines^56^. Five electronic databases (Medline via Ovid, Embase via embase.org, CENTRAL via cochranelibrary.com, CINAHL via EBSCO, and APA PsycInfo via Proquest) were systematically searched for scientific publications on September 2, 2022. The applied search strategy consisted of population-related and intervention-related keywords, developed by Moschonis et al.^19^ and Siopis et al.^20^, combined with a search string to detect economic evaluations, developed by Werbrouck et al.^57^. The latter was originally based on previously published search strings. References^58,59^, but was broadened to maximize sensitivity^57^. The search strategy is further completely consistent with Moschonis et al.^19^ and Siopis et al.^20^ the literature search was restricted by age (adults only), publication date (1 January 2009 onwards to include contemporary evidence only), and language (English), if the search engine allowed to do so. The search strategies for CENTRAL and CINAHL were further restricted to trials only and peer-reviewed manuscripts, respectively. The final search string can be found in Supplementary Methods 1. The search terms and inclusion criteria targeted a broad spectrum of studies with digital components to maximize detection rate. However, only studies with at minimum a smartphone application, text-messaging, or website-based intervention were eventually withheld. Backward and forward citation tracking were performed to identify any studies missed by the search strategy.

As this study is a systematic review, ethical approval was not applicable.

### Study selection and data extraction

Titles and abstracts were screened with Rayyan^60^ by two independent reviewers (RW and NV) based on a priori developed eligibility criteria (Table 3). Importantly, not only head-to-head studies directly comparing the three digital health modes were included, but studies comparing the intervention including a digital health mode to usual care were included as well. Discrepancies were discussed between the two reviewers until consensus was reached. A third reviewer (LA) was available but did not have to step in as there were no discrepancies left.

**Table 3:** Eligibility criteria.

	Inclusion criteria	Exclusion criteria
Population	Adults with or at risk of T2DM and/or hypertension	Children, adolescents, mixed patient populations without stratified results.
Intervention	Smartphone applications, text-messaging, or website interventions	Other digital health interventions (e.g. teleconsultation only, telemonitoring without the use of a smartphone app).
Comparator	Care as usual, face-to-face intervention etc.	N.A.
Outcomes	Full economic outcomes (e.g. cost-effectiveness; cost-utility analysis) or studies capturing both intervention and health resource use costs (cost minimisation).	Outcomes related to either effectiveness, intervention costs, or health resource use costs only.
Publication type and study design	Original research: model-based or within-trial health economic evaluations	Pre/post, reports, systematic reviews, meta-analyses, congress abstracts, protocols, commentaries, animal studies
Language	English	Other languages
Published date	2009 to present	Before 2009

*N.A.* not applicable, *T2DM* type 2 diabetes mellitus.

Eligible full texts were screened by the first author (RW) and one-third of these full texts were screened by a second author (NV). Reference lists from articles that fitted the inclusion criteria were checked for missed articles. The following predetermined data were extracted from all included articles:

- General study characteristics: publication year, country, participant characteristics, intervention alternatives;
- Methods: study perspective (i.e., point of view), economic evaluation type, analytic approach, time horizon (i.e., period of analysis), discount rate (i.e., to convert a value received in the future to a value today), reference year of costs, willingness-to-pay threshold (i.e., what is society prepared to pay for health), intervention costs, health resource use and data sources, information regarding the base case and sensitivity analyses;
- Results and conclusion: (incremental) costs and effects, results from sensitivity analyses, author’s conclusions.

### Quality appraisal

As recommended by van Mastrigt et al.^61^, study quality has been appraised with the Consensus on Health Economic Criteria (CHEC) list^62^, since this checklist enables the assessment of both trial- and model-based economic evaluations^25,61^. The two independent reviewers (RW and NV) followed Werbrouck et al.^57^, who suggested small adaptions to the checklist (e.g., ‘not applicable’ was a valid answer option next to yes or no: for instance, whether or not discounting (item 14) was applied, was only considered applicable if a study’s time horizon was >1 year. Such adaptions resulted in a more valid appraisal of individual studies’ quality)^57^. Discrepancies were discussed by the two reviewers until consensus was reached by specifying assessment criteria.

### Evidence table and analysis

The evidence table summarises study characteristics, treatment alternatives, and results from the incremental base case analyses. Sensitivity analyses are addressed in text. The following methodology applies:

- The treatment in the comparator group has been dichotomised into care as usual (CAU) and enhanced care as usual (CAU + ). In the case of the latter, further description was provided.
- Perspective could either be (i) the public healthcare system perspective (i.e., the third-party payer perspective), (ii) the healthcare payer perspective (i.e., including patient costs next to third-party payer costs), (iii) the societal perspective (i.e. including the payer perspective and costs from productivity losses), (iv) the patient perspective, or (v) the organisational perspective. In the case that the perspective was not explicitly stated, the authors made a judgement. A perspective could also be called ‘limited’: a limited societal perspective may for instance account for non-medical costs (i.e., costs such as transport costs to the hospital, which are costs outside the healthcare sector, but directly relatable to the disease) but not for indirect non-medical costs (i.e., productivity losses due to absenteeism or presenteeism).
- In order to improve the comparability between studies from different countries and different reference years^61^, costs and incremental cost-effectiveness ratios (ICERs)/incremental cost-utility ratios (ICURs) were converted via an online calculator (https://eppi.ioe.ac.uk/costconversion/) to 2022 Euro currency values with Belgium as the reference country, to account for purchasing power parities.

Results were analysed together and per delivery mode, disease, and outcome measure. Moreover, possible cost-effectiveness drivers were explored.

### Reporting summary

Further information on research design is available in the Nature Research Reporting Summary linked to this article.

### Supplementary information

Supplemental Material

Reporting Summary

## Data Availability

Template data collection form and full data extracted from included studies will be made publicly available on The Open Science Framework under ‘DigiCare4You health economics’.
