# Supplementary material for: Cost effectiveness review of text messaging, smartphone application, and website interventions targeting T2DM or hypertension
Source: NPJ Digit Med. 2023 Aug 18;6:150. doi: 10.1038/s41746-023-00876-x (PMC10439143; doi:10.1038/s41746-023-00876-x)
Supplement: Supplementary file 1 — Supplemental Material [file 41746_2023_876_MOESM1_ESM.pdf]

DigiCare4You Consortium

Ruben Willems[1], Lieven Annemans[1], George Siopis[2] [3], George Moschonis[2], Rajesh Vedanthan[4], Jenny Jung[5], Dominika Kwasnicka[6] [7], Brian Oldenburg[6], Claudia d'Antonio[8], Sandro Girolami[8], Eirini Agapidaki[9], Yannis Manios[10] [11], Nick Verhaeghe[1], Natalya Usheva[12], Violeta Iotova[12], Andreas Triantafyllidis[13], Konstantinos Votis[13], Florian Toti[14], Konstantinos Makrilakis[15], Chiara Seghieri[16], Luis Moreno[17], Sabine Dupont[18], Leo Lewis[19], Djordje Djokic[20], Helen Skouteris[21], Enying Gong[5], Bernard Yeboah-Asiamah Asare[5], Vimarsha Kodithuwakku[5], Sofia Segkouli[13], Eleftheria Polychroniou[13], Ioannis Paliokas[13], Odysseas Kyparissis[13], Kostas Anastasiou[10], Konstantinos Tserpes[10], Christina Mavrogianni[10], Eva Karaglani[10], Electra Kalogerakou[10], Maria Maragkoudaki[10], Agathi Ntzouvani[10], Katerina Kontochristopoulou[10], Elizabeth Dupont[18], Leo Dauzon[18], Maartje Roskams[18], Niamh Lennox-Chhugani[19], Edelweiss Aldasoro[19], Nereide A. Curreri[19], Fiona Lyne[19], Darren Curran[19], Pilar Gangas Peiró[19], Alejandro Gil-Salmerón[19], Orla Snook[19], Georgina Ferrer[19], Niamh Daly Day[19], Martin Perrin[19], Nazzareno Pierantozzi[8], Giacomo Vespasiani[8], Teresa Almonti[8], Tracy Taylor[21], Melissa Savaglio[21], George Stergiou[15], Stavros Liatis[15], George Karamanakos[15], Chrysi Koliaki[15], Anastasios Kollias[15], Eva Zikou[15], Haris Dimosthenopoulos[15], Keng-Yen Huang[4], Samrachana Adhikari[4], Kun Qian[4], Julia Dickhaus[4], Kimberly Carney[4], Evette Eweka[4], Farhan Sahito[20], Dusan Pavlovic[20], Arzoo Sahito[20], Gisella Battalova[20], Sabina Nutti[16], Milena Vanieri[16], Nicola Belle[16], Gaia Bertarelli[16], Paola Cantarelli[16], Francesca Ferre[16], Anna Noci[16], Nadia Bozzi[16], Dina Ferrari[16], Rachele Borelli[16], Constanza Tortu[16], Yoto Yotov[12], Kaloyan Tsochev[12], Tanya Stefanova[12], Virginia Atanasova[12], Sonya Koleva[12], Vanya Marinova[12], Vanya Russeva[12], Anna Kozhuharova[12], Rosa Magallón Botaya[17], Gloria Bueno Lozano[17], Pilar De Miguel-Etayo[17], Esther Ma Gonzalez-Gil[17], María L. Miguel-Berges[17], Natalia Giménez-Legarre[17], Bárbara Oliván Blázquez[17], Susana Pérez[17], Skerdi Prifti[14], Blerina Bombaj[14], Luftime Bruka[14], Adriana Lapardhaja[14], Ornela Laze[14], Ditila Doracaj[14].

[1] Interuniversity Center of Health Economic Research (ICHER), department of Public Health and Primary Care, Ghent University, Ghent, Belgium

[2] Department of Food, Nutrition and Dietetics, School of Allied Health, Human Services and Sport, La Trobe University, Melbourne, Australia

[3] Institute for Physical Activity and Nutrition, Deakin University, Geelong, Victoria, Australia

[4] Department of Population Health, NYU Grossman School of Medicine, New York, USA

[5] Maternal, Child and Adolescent Health Program, Burnet Institute, Melbourne, Australia

[6] NHMRC CRE in Digital Technology to Transform Chronic Disease Outcomes, Baker Heart and Diabetes Institute, Melbourne, Australia

[7] Faculty of Psychology, SWPS University of Social Sciences and Humanities, Wroclaw, Poland

[8] Meteda S.r.l., Roma, Italy

[9] Ministry of Health, Athens, Greece

[10] Department of Nutrition and Dietetics, School of Health Science and Education, Harokopio University, Athens, Greece

[11] Institute of Agri-food and Life Sciences, Hellenic Mediterranean University Research Centre, Heraklion, Greece

[12] Medical University of Varna, Varna, Bulgaria

[13] Centre for Research and Technology Hellas – Information Technologies Institute, Hellas, Greece

[14] University of Medicine, Tirana, Albania

[15] National and Kapodistrian University of Athens, Athens, Greece

[16] Sant'Anna School of Advanced Studies, Pisa, Italy

[17] Universidad de Zaragoza, Zaragoza, Spain

[18] International Diabetes Federation European Region, Brussels, Belgium

[19] International Foundation of Integrated Care, Schiphol, The Netherlands

[20] Privanova, Paris, France

[21] Monash University, Melbourne, Australia

Supplementary Methods: Search terms

Medline via OVID

(diabetes OR diabetes mellitus OR type 2 diabetes OR type 2 diabetes mellitus OR type 2 OR type II OR T2D OR TIID OR T2DM OR TIIDM OR prediabetes OR diabetic* OR prediabetic* OR insulin resistance OR insulin resistant OR IR OR insulin sensitivity OR insulin sensitive OR hyperglycaemia OR hyperglycaemic OR hyperglycemia OR hyperglycemic OR blood sugar OR blood glucose OR hypertension OR HT OR HTN OR hypertensive OR blood pressure OR BP OR SBP OR DBP)

AND

(mHealth or mobile health or health app* or digital health or online health or weight loss app* or weight reduction app* or weight management app* or lipid monitoring app* or lipid management app* or glucose monitoring app* or glucose management app* or health monitoring app* or lifestyle app* or diabetes management app* or hypertension management app* or blood pressure management app* or blood pressure monitoring app*)

AND

(cost benefit analysis OR economic evaluation* OR economic analy* OR health economic* OR healtheconomic* OR value for money OR cost analy* OR costanaly* OR cost OR costs OR costly OR costing OR price OR prices OR pricing OR pharmacoeconomic* OR expenditure* OR expense OR expenses OR budget OR budgets OR cost effective OR costeffective OR cost effectiveness OR costeffectiveness OR costefficien* OR costefficien* OR cost benefit* OR costbenefit* OR cost utilit* OR costutilit* OR cost minimization OR costminimization OR quality adjusted life years OR qaly* OR ((quality adjusted OR qualityadjusted) AND (year OR years)))

Narrow by subject age ‘all adults’, published date ‘2009-2022, language ‘English’

= 513 (02/09/2022)

Embase via Embase.org

(diabetes OR 'diabetes mellitus' OR 'type 2 diabetes' OR 'type 2 diabetes mellitus' OR 'type 2' OR 'type ii' OR T2D OR tiid OR t2dm OR tiidm OR prediabetes OR diabetic* OR prediabetic* OR 'insulin resistance' OR 'insulin resistant' OR ir OR 'insulin sensitivity' OR 'insulin sensitive' OR hyperglycaemia OR hyperglycaemic OR hyperglycemia OR hyperglycemic OR 'blood sugar' OR 'blood glucose' OR hypertension OR ht OR htn OR hypertensive OR 'blood pressure' OR bp OR sbp OR dbp)

AND

(mhealth OR 'mobile health' OR 'health app*' OR 'digital health' OR 'online health' OR 'weight loss app*' OR 'weight reduction app*' OR 'weight management app*' OR 'lipid monitoring app*' OR 'lipid management app*' OR 'glucose monitoring app*' OR 'glucose management app*' OR 'health monitoring app*' OR 'lifestyle app*' OR 'diabetes management app*' OR 'hypertension management app*' OR 'blood pressure management app*' OR 'blood pressure monitoring app*' OR telehealth OR telemedicine OR teledietetics OR telediet*)

AND

('cost benefit analysis' OR 'economic evaluation*' OR 'economic analy*' OR 'health economic*' OR healtheconomic* OR 'value for money' OR 'cost analy*' OR costanaly* OR cost OR costs OR costly OR costing OR price OR prices OR pricing OR pharmacoeconomic* OR expenditure* OR expense OR expenses OR budget OR budgets OR 'cost effective' OR costeffective OR 'cost effectiveness' OR costeffectiveness OR costefficien* OR costefficien* OR 'cost benefit*' OR costbenefit* OR 'cost utilit*' OR costutilit* OR 'cost minimization' OR costminimization OR 'quality adjusted life years' OR qaly* OR (('quality adjusted' OR qualityadjusted) AND (year OR years)))

Narrow by subject age ‘all adults’, published date ‘2009-2022, language ‘English’

= 1079 (02/09/2022)

Central via cochranelibrary.com

(diabetes OR "diabetes mellitus" OR "type 2 diabetes" OR "type 2 diabetes mellitus" OR "type 2" OR "type II" OR T2D OR TIID OR T2DM OR TIIDM OR prediabetes OR diabetic* OR prediabetic* OR "insulin resistance" OR "insulin resistant" OR IR OR "insulin sensitivity" OR "insulin sensitive" OR hyperglycaemia OR hyperglycaemic OR hyperglycemia OR hyperglycemic OR "blood sugar" OR "blood glucose" OR hypertension OR HT OR HTN OR hypertensive OR "blood pressure" OR BP OR SBP OR DBP)

AND

(mHealth or "mobile health" or "health app*" or "digital health" or "online health" or "weight loss app*" or "weight reduction app*" or "weight management app*" or "lipid monitoring app*" or "lipid management app*" or "glucose monitoring app*" or "glucose management app*" or "health monitoring app*" or "lifestyle app*" or "diabetes management app*" or "hypertension management app*" or "blood pressure management app*" or "blood pressure monitoring app*")

AND

("cost benefit analysis" OR "economic evaluation*" OR "economic analy*" OR "health economic*" OR healtheconomic* OR "value for money" OR "cost analy*" OR costanaly* OR cost OR costs OR costly OR costing OR price OR prices OR pricing OR pharmacoeconomic* OR expenditure* OR expense OR expenses OR budget OR budgets OR "cost effective" OR costeffective OR "cost effectiveness" OR costeffectiveness OR costefficien* OR costefficien* OR costefficien* OR "cost benefit*" OR costbenefit* OR "cost utilit*" OR costutilit* OR "cost minimization" OR costminimization OR "quality adjusted life years" OR qaly* OR "quality adjusted year*" OR "qualityadjusted year*")

Narrow by published date '2009-2022', methodology 'trials'.

= 154 (02/09/2022)

Cinahl via EBSCO

(diabetes OR "diabetes mellitus" OR "type 2 diabetes" OR "type 2 diabetes mellitus" OR "type 2" OR "type II" OR T2D OR TIID OR T2DM OR TIIDM OR prediabetes OR diabetic* OR prediabetic* OR "insulin resistance" OR "insulin resistant" OR ir OR "insulin sensitivity" OR "insulin sensitive" OR hyperglycaemia OR hyperglycaemic OR hyperglycemia OR hyperglycemic OR "blood sugar" OR "blood glucose" OR hypertension OR HT OR HTN OR hypertensive OR "blood pressure" OR BP OR SBP OR DBP)

AND

((MHealth or (MH "Telehealth+")) or (health and ((app* or (MH "Mobile Applications") or mobile or digital or online)) or telemedicine or telediet*)

AND

("cost benefit analysis" OR "economic evaluation*" OR "economic analy*" OR "health economic*" OR healtheconomic* OR "value for money" OR "cost analy*" OR costanaly* OR cost OR costs OR costly OR costing OR price OR prices OR pricing OR pharmacoeconomic* OR expenditure* OR expense OR expenses OR budget OR budgets OR "cost effective" OR costeffective OR "cost effectiveness" OR costeffectiveness OR costefficien* OR costefficien* OR "cost benefit*" OR costbenefit* OR "cost utilit*" OR costutilit* OR "cost minimization" OR costminimization OR "quality adjusted life years" OR qaly* OR (("quality adjusted" OR qualityadjusted) AND (year OR years)))

Narrow by subject age 'all adults', published date '20090101-20221231', language 'English', 'peer reviewed'

= 1303 (02/09/2022)

APA PsycInfo Search via proquest

noft((MHealth OR telehealth OR (health AND (app* OR mobile OR digital OR online)) OR telemedicine OR telediet* OR "weight loss app*" OR "weight reduction app*" OR "weight management app*" OR "lipid monitoring app*" OR "lipid management app*" OR "glucose monitoring app*" OR "glucose management app*" OR "health monitoring app*" OR "lifestyle app*" OR "diabetes management app*" OR "hypertension management app*" OR "blood pressure management app*" OR "blood pressure monitoring app*")

AND

(diabetes OR "diabetes mellitus" OR "type 2 diabetes" OR "type 2 diabetes mellitus" OR "type 2" OR "type II" OR T2D OR TIID OR T2DM OR TIIDM OR prediabetes OR diabetic* OR prediabetic* OR "insulin resistance" OR "insulin resistant" OR ir OR "insulin sensitivity" OR "insulin sensitive" OR hyperglycaemia OR hyperglycaemic OR hyperglycemia OR hyperglycemic OR "blood sugar" OR "blood glucose" OR hypertension OR HT OR HTN OR hypertensive OR "blood pressure" OR BP OR SBP OR DBP)

AND

("cost benefit analysis" OR "economic evaluation*" OR "economic analy*" OR "health economic*" OR healtheconomic* OR "value for money" OR "cost analy*" OR costanaly* OR cost OR costs OR costly OR costing OR price OR prices OR pricing OR pharmacoeconomic* OR expenditure* OR expense OR expenses OR budget OR budgets OR "cost effective" OR costeffective OR "cost effectiveness" OR costeffectiveness OR costefficien* OR costefficien* OR "cost benefit*" OR costbenefit* OR "cost utilit*" OR costutilit* OR "cost minimization" OR costminimization OR "quality adjusted life years" OR qaly* OR "quality adjusted year*" OR "qualityadjusted year*"))

Narrow by Date ‘After December 31 2008’, Language ‘English’, Age group ‘Adulthood (18 Yrs & Older)’

=7 (02/09/2022)

Supplementary Methods 2: Item assessment instructions

1	Study population	The relevant clinical characteristics, entry and eligibility criteria, as well as drop-out during follow-up should be stated explicitly. If model-based: population should be described in function of the model: e.g., prevalence/incidence, life expectancy (instead of eligibility criteria, etc).
2	Competing alternatives	A detailed description should be given of the competing interventions. This should encompass a clear and specific statement of the primary objective of each alternative, as well as relevant factors, such as intensity, duration, and frequency. Only ‘care as usual’ for the competing alternative is not enough.
3	Research question	A research question has to identify clearly the alternatives being compared and the population for which the comparison is made. It should include a description of the population, the intervention and the control group treatment, and the aim (outcome).
4	Study design	An appropriate economic study design is a full economic evaluation (comparison of costs and health consequences of two or more interventions) based on primary research (cohort, case-control, randomised controlled trial).
5	Time horizon	The period of analysis of the study is the time horizon. This time horizon should always be equal for costs and outcomes if these are combined in a ratio. The time span should be long enough to include all relevant costs and outcomes relating the intervention. Ideally, the follow-up period should be extended till the situation is stabilised with reference to costs and effects. Since T2DM (and HT) are considered chronic diseases, an arbitrary time horizon of minimum 10 years is considered long enough.
6	Perspective	‘Perspective’ indicates from which point of view an economic evaluation study is performed. If the study is performed from a societal perspective tick ‘yes’, as all relevant costs and consequences of an interventions and disease are taken into account, if possible. Other narrower perspectives will only include certain components. The authors should motivate why a narrower perspective is valid.
7	Costs: identification	A full identification of all important and relevant costs should be given in relation to the perspective and the research question.
8	Costs: measurement	The costs should be measured appropriately in physical units. The instrument by which the costs are measured should be valid and clearly stated (e.g. interview, questionnaire, cost-diary).
9	Costs: value	The sources of valuation should be clearly stated for each cost price of every volume parameter and their reference year. The main cost should be calculated based on depleted sources, no tariffs should be used.

10	Outcomes: identification	A full identification of all important and relevant outcomes should be given in relation to the perspective and the research question.
11	Outcomes: measurement	The outcome measurement should result from the outcome identification and this should be straightforward (e.g. if mortality is a main outcome measure this should be taken into account in the analysis). The instrument by which the outcomes are measured should be valid and clearly stated.
12	Outcomes: value	The method of outcome valuation should be clearly stated. Examples of valuation methods are Discrete Choice Experiments (e.g. Conjoint analysis, Contingent valuation), Direct utility assessment (VAS, TTO, SG, etc.), Indirect utility assessment (HUI, EQ-5D, QWB, etc.), Person trade off, etc.
13	Incremental analysis	An incremental analysis should examine the additional costs from one intervention over another, compared to the additional outcomes that it delivers. The incremental costs-effectiveness ratio is obtained by dividing the costs differences by the outcome differences for the alternatives. Not applicable if item 4 is scored 0.
14	Discounted	Discounting is done appropriately if all costs and outcomes are converted to one single year, based on a motivated discount rate. Not applicable is the time horizon is <1 year.
15	Sensitivity analysis	All variables in the analysis are potential candidates for the sensitivity analysis. Only variables that are certain or which have a minimal impact on the study results (based on the preliminary analysis) can be excluded from the sensitivity analysis. Furthermore, a justification should be given over the range of the variables used in the sensitivity analysis. Minimum PSA or bootstrapping AND another kind of sensitivity analysis needed.
16	Conclusions	Do the authors interpret their results cautiously and are their conclusions justified by the data.
17	Generalizability	This can be done by being explicit about the viewpoint of analysis and by indicating how particular costs and outcomes vary by location, setting, patient population, care provider, etc.
18	No conflict of interest	Not stated or conflict of interest: score 0.
19	Ethics	Does the article notes ethical aspects and elaborates on the characteristics of the population experiencing the disease or the intervention (young, old, poor, wealthy) and how this may have distributional implications.

Supplementary note 1

List of excluded studies at full text screening and reason why

Reference	Exclusion reason
Augustovski, F., et al. (2017). "Cost-effectiveness analysis of a randomized trial of an mhealth intervention to improve cardiometabolic profile in prehypertensive subjects from low-resource urban settings in Latin America." Value in Health 20(9): A916.	Conference abstract
Berg, G. D. and S. Wadhwa (2009). "Diabetes disease management results in Hispanic Medicaid patients." Journal of Health Care for the Poor & Underserved 20(2): 432-443.	Population
Bohingamu Mudiyanselage, S., et al. (2019). "Personalised telehealth intervention for chronic disease management: A pilot randomised controlled trial." Journal of Telemedicine and Telecare 25(6): 343-352.	Population
Bovbjerg, M. L., et al. (2017). "Pilot study for managing complex chronic care medicaid patients with diabetes using a mobile health application achieves "triple aim" improvement in a primary care setting." Clinical Diabetes 35(4): 227-231.	Population
Cárdenas, M. K., et al. (2015). "The cost of illness attributable to diabetic foot and cost-effectiveness of secondary prevention in Peru." BMC Health Services Research 15: 1-10.	Intervention
Crowley, M. J., et al. (2022). "Effect of a Comprehensive Telehealth Intervention vs Telemonitoring and Care Coordination in Patients with Persistently Poor Type 2 Diabetes Control: A Randomized Clinical Trial." JAMA Internal Medicine.	Intervention
Datta, S. K., et al. (2010). "Economic analysis of a tailored behavioral intervention to improve blood pressure control for primary care patients." American Heart Journal 160(2): 257-263.	Intervention
Dehmer, S. P., et al. (2018). "Economic evaluation of the home blood pressure telemonitoring and pharmacist case management to control hypertension (Hyperlink) trial." JACCP Journal of the American College of Clinical Pharmacy 1(1): 21-30.	Intervention
Detournay, B., et al. (2020). "Diabeo system with and without telemonitoring could be associated with lower diabetes management costs versus standard care in poorly controlled diabetic patients." Diabetes Technology and Therapeutics 22: A-151.	Conference abstract
Egede, L. E., et al. (2018). "Cost-effectiveness of technology-assisted case management in low income adults with diabetes." Journal of General Internal Medicine 33(2): 150-151.	Intervention
Fottrell, E., et al. (2019). "Community groups or mobile phone messaging to prevent and control type 2 diabetes and intermediate hyperglycaemia in Bangladesh (DMagic): a cluster-randomised controlled trial." The lancet. Diabetes & endocrinology 7(3): 200-212.	Outcome
Frederix, I., et al. (2016). "Effect of comprehensive cardiac telerehabilitation on one-year cardiovascular rehospitalization rate, medical costs and quality of life: A cost-effectiveness analysis." European Journal of Preventive Cardiology 23(7): 674-682.	Population
Gordon, L. G., et al. (2014). "A cost-effectiveness analysis of a telephone-linked care intervention for individuals with Type 2 diabetes." Diabetes Research and Clinical Practice 104(1): 103-111.	Intervention
Graves, N., et al. (2009). "Cost-Effectiveness of a Telephone-Delivered Intervention for Physical Activity and Diet." PLoS ONE 4(9): e7135.	Intervention
Hay, J. W., et al. (2018). "Cost-Effectiveness of a Technology-Facilitated Depression Care Management Adoption Model in Safety-Net Primary Care Patients with Type 2 Diabetes." Value in Health 21(5): 561-568.	Outcome
Henderson, C., et al. (2013). "Cost effectiveness of telehealth for patients with long term conditions (Whole Systems Demonstrator telehealth questionnaire study): Nested economic evaluation in a pragmatic, cluster randomised controlled trial." BMJ (Online) 346(7902).	Population
Ionov, M., et al. (2017). "Telemonitoring and remote counseling in hypertensive patients: A route to cost effectiveness." Journal of Hypertension 35: e255-e256.	Conference abstract
Ionov, M., et al. (2021). "Blood pressure telemonitoring and remote counseling in patients with uncontrolled hypertension: A cost-utility analysis." Journal of Hypertension 39(SUPPL 1): e217-e218.	Language

Islam, S. M. S. (2018). "Effectiveness and cost effectiveness of a mobile phone text messaging intervention for prevention of cardiovascular risk factors among patients with type 2 diabetes: A randomized controlled trial." Journal of Hypertension 36: e315.	Conference abstract
Kaambwa, B., et al. (2014). "Telemonitoring and self-management in the control of hypertension (TASMINH2): a cost-effectiveness analysis." European Journal of Preventive Cardiology 21(12): 1517-1530.	Intervention
Laiteerapong, N., et al. (2018). "Individualized Glycemic Control for U.S. Adults With Type 2 Diabetes: A Cost-Effectiveness Analysis." Annals of Internal Medicine 168(3): 170-178.	Intervention
Levin, K., et al. (2013). "Telemedicine diabetes consultations are cost-effective, and efects on essential diabetes treatment parameters are similar to conventional treatment: 7-year results from the Svendborg telemedicine diabetes project." Journal of Diabetes Science and Technology 7(3): 587-595.	Outcome
Madsen, L. B., et al. (2011). "Economic evaluation of home blood pressure telemonitoring: a randomized controlled trial." Blood Pressure 20(2): 117-125.	Intervention
Main, F., et al. (2021). "Technology-enabled remote management of diabetes foot disease and potential for reduction in associated health costs: a pilot study." Journal of foot and ankle research 14(1): 7.	Population
McKnight, T., et al. (2018). "Assessing Effectiveness and Cost-Benefit of the Trinity Hospital Twin City Fit For Life Program for Weight Loss and Diabetes Prevention in a Rural Midwestern Town." Preventing Chronic Disease 15: 1-13.	Intervention
Murry, L. T., et al. (2020). "Evaluation of a clinical pharmacist team-based telehealth intervention in a rural clinic setting: A pilot study of feasibility, organizational perceptions, and return on investment." Pilot and Feasibility Studies 6(1).	Design
Neumann, A., et al. (2017). "The cost-effectiveness of interventions targeting lifestyle change for the prevention of diabetes in a Swedish primary care and community based prevention program." European Journal of Health Economics 18(7): 905-919.	Intervention
Niu, B., et al. (2021). "Cost-effectiveness of telehealth with remote patient monitoring for postpartum hypertension." Journal of Maternal-Fetal and Neonatal Medicine.	No full text
Nobis, S., et al. (2018). "Web-based intervention for depressive symptoms in adults with types 1 and 2 diabetes mellitus: A health economic evaluation." British Journal of Psychiatry 212(4): 199-206.	Population
Nundy, S., et al. (2014). "Mobile phone diabetes project led to improved glycemic control and net savings for chicago plan participants." Health Affairs 33(2): 265-272.	Population
Oksman, E., et al. (2017). "Cost-effectiveness analysis for a tele-based health coaching program for chronic disease in primary care." BMC Health Services Research 17(1): 138.	Intervention
O'Reilly, D. J., et al. (2022). "Economic Analysis of a Diabetes Health Coaching Intervention for Adults Living With Type 2 Diabetes: A Single-Centre Evaluation From a Community-Based Randomized Controlled Trial." Canadian Journal of Diabetes 46(2): 165-170.	Intervention
Pozo-Martin, F., et al. (2021). "Cost-effectiveness of a Community-based Hypertension Improvement Project (ComHIP) in Ghana: Results from a modelling study." BMJ Open 11(9).	Intervention
Smith, K. J., et al. (2016). "Cost effectiveness of an internet-delivered lifestyle intervention in primary care patients with high cardiovascular risk." Preventive Medicine 87: 103-109.	Population
Varney, J. E., et al. (2016). "The cost-effectiveness of hospital-based telephone coaching for people with type 2 diabetes: a 10 year modelling analysis." BMC Health Services Research 16(1): 521.	Intervention
Wang, V., et al. (2012). "Economic evaluation of telephone self-management interventions for blood pressure control." American Heart Journal 163(6): 980-986.	Intervention
Warren, R., et al. (2018). "Effects of telemonitoring on glycaemic control and healthcare costs in type 2 diabetes: A randomised controlled trial." Journal of Telemedicine and Telecare 24(9): 586-595.	Intervention
Yang, L., et al. (2022). "Effects of Mobile Phone–Based Telemedicine Management in Patients With Type 2 Diabetes Mellitus: A Randomized Clinical Trial." American journal of the medical sciences 363(3): 224-231.	No full text
